# Expression of FGF8, FGF18, and FGFR4 in Gastroesophageal Adenocarcinomas

**DOI:** 10.3390/cells8091092

**Published:** 2019-09-16

**Authors:** Gerd Jomrich, Xenia Hudec, Felix Harpain, Daniel Winkler, Gerald Timelthaler, Thomas Mohr, Brigitte Marian, Sebastian F. Schoppmann

**Affiliations:** 1 Department of Surgery, Medical University of Vienna and Gastroesophageal Tumor Unit, Comprehensive Cancer Center (CCC), Spitalgasse 23, 1090 Vienna, Austria; gerd.jomrich@meduniwien.ac.at (G.J.); felix.harpain@meduniwien.ac.at (F.H.); sebastian.schoppmann@meduniwien.ac.at (S.F.S.); 2 Department of Medicine I, Institute of Cancer Research, Medical University of Vienna, Borschkegasse 8a, 1090 Vienna, Austria; xenia.hudec@meduniwien.ac.at (X.H.); gerald.timelthaler@meduniwien.ac.at (G.T.); thomas.mohr@meduniwien.ac.at (T.M.); 3 Department of Statistics and Operations Research, University of Vienna, Oskar Morgenstern Platz 1, 1090 Vienna, Austria; Daniel.Winkler@wu.ac.at

**Keywords:** FGF8, FGF18, FGFR4, adenocarcinoma of the esophagogastric junction, neoadjuvant therapy

## Abstract

Even though distinctive advances in the field of esophageal cancer therapy have occurred over the last few years, patients’ survival rates remain poor. FGF8, FGF18, and FGFR4 have been identified as promising biomarkers in a number of cancers; however no data exist on expression of FGF8, FGF18, and FGFR4 in adenocarcinomas of the esophago-gastric junction (AEG). A preliminary analysis of the Cancer Genome Atlas (TCGA) database on FGF8, FGF18, and FGFR4 mRNA expression data of patients with AEG was performed. Furthermore, protein levels of FGF8, FGF18, and FGFR4 in diagnostic biopsies and post-operative specimens in neoadjuvantly treated and primarily resected patients using immunohistochemistry were investigated. A total of 242 patients was analyzed in this study: 87 patients were investigated in the TCGA data set analysis and 155 patients in the analysis of protein expression using immunohistochemistry. High protein levels of FGF8, FGF18, and FGFR4 were detected in 94 (60.7%), 49 (31.6%) and 84 (54.2%) patients, respectively. Multivariable Cox proportional hazard regression models revealed that high expression of FGF8 was an independent prognostic factor for diminished overall survival for all patients and for neoadjuvantly treated patients. By contrast, FGF18 overexpression was significantly associated with longer survival rates in neoadjuvantly treated patients. In addition, FGF8 protein level correlated with Mandard regression due to neoadjuvant therapy, indicating potential as a predictive marker. In summary, FGF8 and FGF18 are promising candidates for prognostic factors in adenocarcinomas of the esophago-gastric junction and new potential targets for new anti-cancer therapies.

## 1. Introduction

Esophageal Cancer (EC) is the eighth most common cancer worldwide. Whereas the number of esophageal squamous cell carcinomas (ESCC) is decreasing, the number of adenocarcinomas of the esophago-gastric junction (AEG) is increasing dramatically [1]. Despite improvements in diagnostics and the use of multimodal approaches, combining surgical resection with perioperative chemo-(radio) therapy, overall prognosis of AEG remains poor [2,3]. Survival rates vary considerably among patients with AEG, and an appreciable proportion of patients with advanced stages develop recurrence, even after initially curative resection [4,5]. Therapy response is often limited due to a number of inherent mechanisms of resistance [6]. This problem is aggravated by the heterogeneity in malignant tumors, containing a small subpopulation of cancer stem-like cells (CSC), characterized by a long lifespan and enhanced survival capacity that supports drug resistance [7,8]. Stem cell characteristics of CSCs are governed by the activity of distinct stem cell specific regulatory pathways leading to cancer relapse as well as chemo- and radio-resistance [9]. The role of CD133- and CD44-positive subpopulation in EC has been described recently [10,11,12] and the wnt-, notch-, hedgehog-, and hippo-pathways have been identified as stem cell specific targets driving therapy resistance and relapse [10,11,12,13]. Both CSC-specific signaling pathways and the survival capacity of a larger tumor cell pool might influence the therapy response.

Specifically, FGFs have found their way into anti-cancer therapy as targets to overcome resistance to chemotherapy in a number of different malignancies [14]. FGFs play a major physiological role in embryonic development and tissue repair by mediating strong survival signals via activation of the direct receptor substrate FRS2α, and the RAS- and PI3K-pathways [15,16,17]. In cancers the pathway might be deregulated by manifold-mechanisms causing either hyperactivation or even constitutively active FGFR-dependent survival signaling [14]. Both expression of specific receptors and up-regulation of autocrine FGF ligands have been found to be associated with resistance to chemo-(radiation) as well as to targeted therapy [18,19,20,21,22]. Previously, our group has studied a CD44-positive stem-like population in colorectal cancer (CRC) and identified a wnt-driven FGF18-dependent autocrine-signaling loop as a strong driver of tumor cell survival [8,23]. Furthermore, we demonstrated a progressive up regulation of FGF18 in CRC [24]. The growth factor induces autocrine survival signaling via the FGF receptor FGFR3-IIIc and blocking of this receptor inhibits tumor growth by inducing apoptosis [25]. Alternatively, FGF18 effects may be mediated by FGFR4, a receptor for which a polymorphic variant exists that causes substitution of an arginine for a glycine at position 388 in the transmembrane domain [26,27,28]. FGF8 is known to play an important role in embryonic development [29,30]. In tumors, overexpression of FGF8 is associated with diminished survival based on stimulating anti-apoptotic pathways mediated by the IIIc splice variants of FGFR1, 2, 3 as well as FGFR4 [28,31,32]. Recently, we could show that the expression of FGF8 was strongly associated with the regression grade in neoadjuvantly treated colorectal cancer patients [33].

Until now, little has been known about the role of FGFs and their receptors in AEG, in particular to the best of our knowledge no data was published describing the expression of FGF 8, 18, and FGFR4 in AEG. Therefore, the aim of this study is to investigate the role of FGF 8, 18, and FGFR4 in AEG in order to define predictive markers and possibly identify suitable new targets for multimodal therapies.

## 2. Materials and Methods

### 2.1. Preliminary TCGA (The Cancer Genome Atlas) Analysis

Data (HTSeq counts) for AEG were downloaded from the TCGA-ESCA project, preprocessed, and normalized using the TCGABiolinks package of R [34]. Optimal cutoff values for gene expression were determined by maximizing the log-rank statistics using the survminer package of R [35]. Differentially expressed genes where determined using TCGABiolinks, employing the edgeR algorithm with exact testing [36]. Gene expression of relevant KEGG pathways was visualized using pathview [37,38].

### 2.2. Patient Selection

Patients who underwent a resection of gastroesophageal adenocarcinomas between January 1992 and April 2012 at the Department of Surgery at the Medical University Vienna were identified from a prospectively maintained database. Patients with distant metastasis at time of diagnosis were excluded. The study was approved by the Ethics Committee of the Medical University of Vienna, Austria, according to the declaration of Helsinki (EK 1652/2016). Patients with locally advanced AEG received neoadjuvant chemotherapy according to the recommendation of the interdisciplinary tumor board meeting. Regression grade to neoadjuvant chemotherapy was classified as defined by Mandard A.M. et al. [39]. The tumor stage was conducted according to the pathological tumor-node-metastasis (TNM) classification of the Union for International Cancer Control (UICC), 7th edition.

### 2.3. Immunohistochemistry

Immunohistochemistry (IHC) was performed on paraffin-embedded specimens fixed in 4% buffered formalin, using 3-µm-thick histological sections. Furthermore, per case two tissue cylinders with a 2.0 millimeter diameter were punched from representative tissue areas to build a tissue micro array (TMA), as described previously [40]. Expression of FGF8, FGF18, and FGFR4 was detected by using polyclonal rabbit antibodies as follows: FGF8 antibody (Abcam, Cambridge, UK, ab203030) in a dilution of 1:600, FGF18 antibody (Assay Biotech, Fremont, CA, USA, C12364) in a dilution of 1:500, and FGFR4 antibody (Santa Cruz Biotechnology, Dallas, TX, USA, sc-124) in a dilution of 1:400, respectively. Secondary antibody was biotinylated and coupled to an avidin-biotin-HRP complex (Thermo Scientific™ Lab Vision™ UltraVision™ LP, Waltham, MA, USA). 3,30-diaminobenzidine (DAB; Chromogen) was used to visualize the staining and counterstaining was achieved with hematoxylin. Antibodies used in this study were optimized for gastroesophageal adenocarcinomas on colorectal cancer tissue with known expression from previously published studies [33,41]. Two observers (J.G. and H.F.) independently reviewed all slides. For the quantitative evaluation of expression, only epithelial cells were investigated. Immunostaining scores (0–12) of FGF8, FGF18, and FGFR4 were calculated as the products of the staining intensity (0 = negative, 1 = weak, 2 = moderate or 3 = strong expression) and points (0–4) were given for the percentages of tumor cells showing positive staining 0 (<1%), 1 (1–10%), 2 (10–50%), 3 (51–80%), and 4 (>80%). Tumors were considered to have high expression with final scores exceeding the median score. Tumors showing expression equal or below the median were considered as being low or absent.

### 2.4. Statistical Analysis

Statistical analysis was performed using the R Statistical Software, Vienna, Austria (Version 3.6) with the “survival” package [42,43]. Univariable and multivariable analyses were conducted using the Cox proportional hazard model. The graphical analysis was performed using the Kaplan-Meier estimator. Plotting was performed using the “survminer” package [35]. The significance of differences in survival times were determined with a log-rank test. Correlations between clinicopathological parameters and FGF8, FGF18, and FGFR4 expression levels were analyzed with the x^2^ test. In order to measure statistical dependence between FGF 8 and FGF 18 the non-parametric Kendall’s rank correlation was used.

Overall survival (OS) was defined as the time between surgery and the patients’ death. Death from causes other than AEG or survival until the end of the observation was considered as censored observations.

## 3. Results

### 3.1. Preliminary TCGA (The Cancer Genome Atlas) Analysis

**Figure 1:**
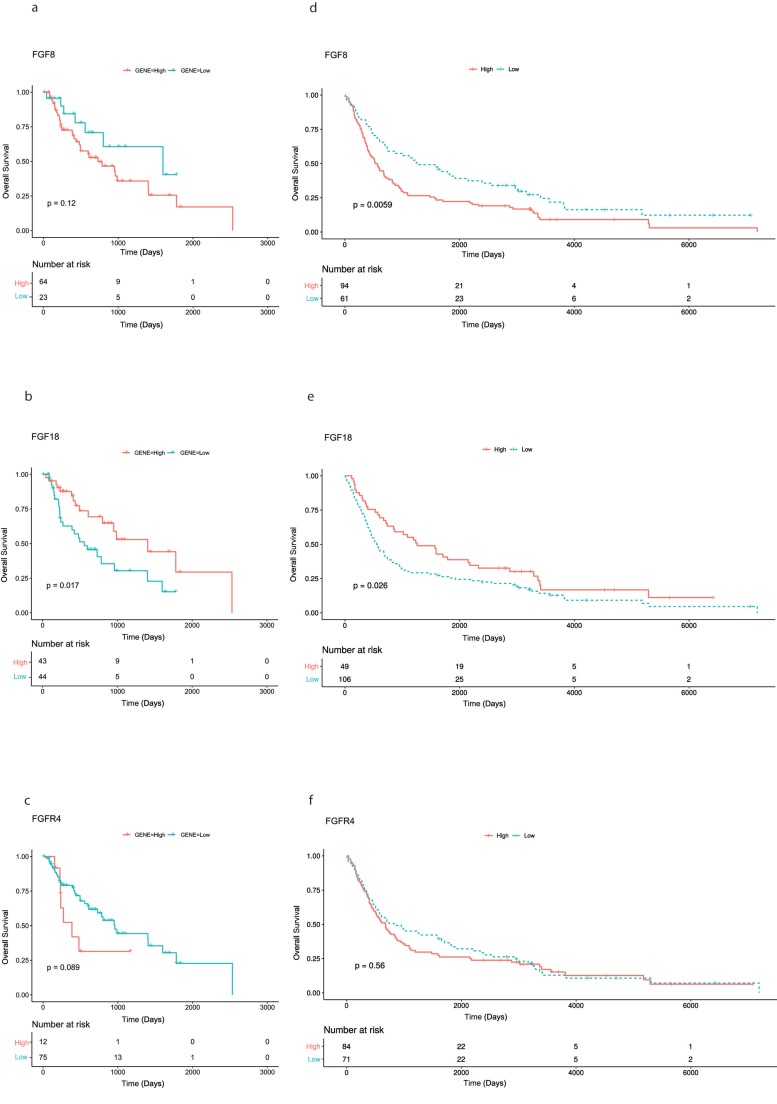
Kaplan-Meier curves of overall survival of patients with adenocarcinomas of the esophago-gastric junction. (**a**–**c**) Patients from TCGA data set analysis: high FGF8, FGF18, and FGFR4 expression compared with those with low/absent FGF8, FGF18, and FGFR4 expression. (**d**–**f**) Patients from the immunohistological analysis: high FGF8, FGF18, and FGFR4 expression compared with those with low/absent FGF8, FGF18, and FGFR4 expression.

While investigating mRNA expression data of patients with AEG (n = 87) available from the TCGA data base, overexpression of FGF8, FGF18, and FGFR4 was found in 64, 43, 12 cases, respectively. No significant correlation of overexpression of FGF8, FGF18, and FGFR4 and clinicopathological parameters (tumor stage, lymph node status and age) was found. Survival analysis using Kaplan-Meier curves for visualization, found significantly better OS rates for patients with FGF18 overexpressing tumors (*p* = 0.017). No significance could be found for FGF8 and FGFR4 (Figure 1a–c).

### 3.2. Immunohistochemical Analysis of Tumor Tissue Samples

**Figure 2:**
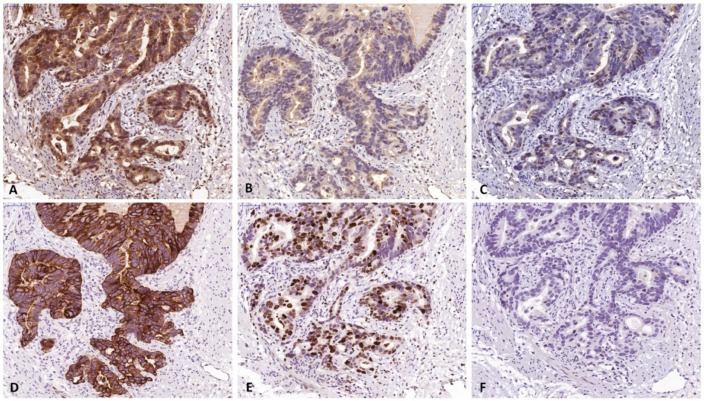
Specimen of adenocarcinomas of the esophago-gastric junction stained for (**a**) FGF8, (**b**) FGF18 and (**c**) FGFR4. Positive staining was found in the tumor cells and to a lesser degree in the microenvironment. FGF8 and FGFR4 expression were primarily found in the nucleus, while FGF18 expression was mainly found in the cytoplasm. For quantitative evaluation, only epithelial cells were investigated. Corresponding sections stained by CK7 (**d**), Ki67 (**e**), and negative control (**f**). (The bar corresponds to 50 µm.) Original magnification ×400 all).

**Figure 3:**
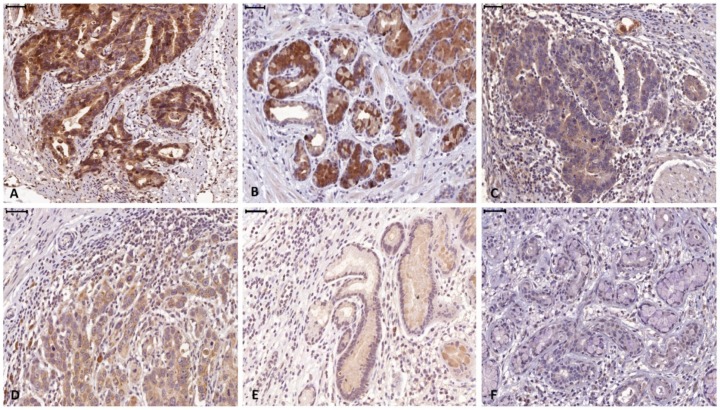
Representative high (**a**–**c**) and low (**d**–**f**) expressing tumor section of FGF8 (**a** and **d**), FGF18 (**b** and **e**), and FGFR4 (**c** and **f**).

A total of 155 patients (124 males, 80%) with histologically verified AEG were investigated for this study. From 10 patients full section slides were investigated to confirm staining quality for all antibodies used in this study. Tissue specimens of the tumors were stained for FGF8, FGF18, FGFR4, cytokeratin 7 (CK7) and the proliferation marker Ki67 (Figure 2). For all 3 markers staining was predominantly seen in the cytoplasm of tumor cells. Weaker staining was also observed in the tumor stroma. For quantification, only tumor cell staining was assessed. High expression of FGF8, FGF18, and FGFR4 was found in 94 (60.7%), 49 (31.6%) and 84 (54.2%), respectively (Figure 3a–c) as compared to low expressing areas (Figure 3d–f). Each marker had a distinct expression pattern with no correlation between individual markers. Correlation of clinicopathological parameters and expression of FGF8, FGF18, and FGFR4 in the tumor tissue revealed significant correlations of the FGF8 protein level with tumor size ((y)pT), UICC stage, and Mandard regression grade (Table 1). FGFR4 protein level only correlates with gender and for FGF18 no relationship with any clinical parameter could be observed (compiled in Table 1).

69 (44.5%) patients received neoadjuvant treatment. Median time of OS was 23 months (range 0.3–236.0 months) and 134 patients died during the time of observation. The rate of 3- and 5-year OS was 38.7% and 29.7%, respectively. However, Kaplan-Meier analysis shows a significant correlation between high FGF8 expression (*p* = 0.006) and reduced patients’ OS, high FGF18 expression (*p* = 0.026) was significantly associated with longer patients’ OS. No significance was found for FGFR4 expression and patients’ survival (Figure 1d–f).

Univariable Cox proportional hazard regression revealed that high expression of FGF8 (HR 0.61, 95% CI 0.43-0.87, *p* = 0.006), advanced tumor stage (HR 2.74, 95% CI 1.37–5.50, *p* = 0.005), poor tumor differentiation (HR 0.57, 95% CI 0.39–0.83, *p* = 0.003), high lymph node ratio (HR 1.93, 95% CI 1.35–2.77, *p* < 0.001), positive resection margin (HR 2.06, 95% CI 1.36–3.10, *p* < 0.001), and receiving adjuvant treatment (HR 1.55, 95% CI 1.10–2.18, *p* = 0.013) were significantly associated with impaired patients OS, whereas high expression of FGF18 (HR 0.6, 95% CI 0.45-0.95, *p* = 0.027), negative lymph node status (HR 0.23, 95% CI 0.14–0.39, *p* < 0.001), and low UICC staging (HR 0.34, 95% CI 0.19–0.63, *p* < 0.001), were significantly associated with improved OS (Table 2). Univariable subgroup analysis revealed significant correlation for tumor size, lymph node status and UICC stage, and OS in both neoadjuvantly treated and primarily resected patients. Tumor differentiation and resection margin were found to be significantly associated with OS only in neoadjuvantly treated patients and lymph node ratio only in primarily resected patients (Table 2). Further results of univariable subgroup analysis of neoadjuvantly treated and primarily resected patients can be found in Table 2 as well.

For multivariable Cox proportional hazard regression analysis separate models for FGF8, FGF18, and FGFR4 were used. Besides FGF8, FGF18, and FGFR4 the factors age, gender, tumor differentiation, UICC stage, lymph node ratio, adjuvant treatment, and Mandard regression grade (in neoadjuvantly treated patients only) were included. In multivariable analysis, high FGF8 (HR 0.68, 95% CI 0.46–0.99, *p* = 0.04) was identified as the only independent predictor for shorter OS. Subgroup analysis of neoadjuvantly treated and primarily resected patients revealed that high FGF8 (HR 0.43, 95% CI 0.22–0.82, *p* = 0.011) and FGF18 (HR 0.44, 95% CI 0.22–0.86, *p* = 0.017) in neoadjuvantly treated but not in primarily resected patients remained as independent predictors for OS (Table 3).

## 4. Discussion

Despite significant improvements in diagnosis, surgical techniques and multimodal perioperative therapies over the last few years, survival rates of patients suffering from adenocarcinoma of the esophago-gastric junction remain poor. To investigate and understand the pathophysiological mechanisms of tumorigenesis in these cancers might be the key to better therapies and therefore to improved survival rates.

In this study, we show the utility of FGF8 and FGF18 as independent prognostic markers in AEG for the first time.

FGF and FGFR as targets have recently found their way into anti-cancer therapy, especially to overcome chemo- and radio-resistance [14]. Recently, our group investigated the role of FGF8, FGF18, and FGFR4 in colo-(rectal) and hepatocellular cancer [23,24,31,41,44]. However, data of FGF and FGFR expression in adenocarcinomas of the esophago-gastric junction were limited until now. Therefore, a TCGA analysis and immunohistochemistry, including tumor tissue from patients before and after neoadjuvant treatment, was performed to investigate the prognostic role of expression of FGF8, FGF18, and FGFR4 in adenocarcinomas of the esophago-gastric junction.

The analysis revealed significantly shorter OS when tumors were highly abundant in FGF8 in the cohort of all patients (*p* = 0.04) and the subgroup of neoadjuvantly treated patients (*p* = 0.011). This goes in good accordance with published data: overexpression of androgen related FGF8 is known to play a crucial role in prostate and colorectal cancer. Furthermore, Harpain et al. recently found that FGF8 induced therapy resistance in neoadjuvantly treated rectal cancer patients [33,45]. In this study FGF8 expression was found to correlate with Mandard regression grade, suggesting a role of the growth factor in therapy response. Investigating this correlation in a larger cohort would be of high interest.

With regard to FGF18, our observations in AEG contradict older reports: our previously published findings on FGF18 expression demonstrated increased tumor cell survival and migration caused by FGF18 expression in colorectal cancer [25]. In gastric cancer cell lines, Zhang et al. published data of poor survival when tumors were high in FGF18 and related it this to ERK-MAPK signaling [23,24,46]. In contrast to these results, our data show a significantly improved OS in neoadjuvantly treated patients with adenocarcinomas of the esophago-gastric junction when the tumors are high in FGF18. Analysis of the TCGA data set of AEG patients showed similar results, supporting the conclusion that FGF18 is a positive prognostic factor. Previously, we published data on ETV1 and MK2 expression in adenocarcinomas of the esophago-gastric junction, showing that nuclear overexpression of ETV1 was associated with significantly better patients OS [40]. A potential explanation for the protective role of ETV1 and FGF18 overexpression might be found in the ERK-MAPK signaling pathway as mentioned above. However, our findings remain controversial and need further investigation.

Among all the samples tested, overexpression of FGF8 and FGF18 was found in 31 (20%) cases. However, analysis of a potential correlation between FGF8 and FGF18 overexpression in tumor tissue found no significant correlation between these two markers (Kendall’s rank correlation).

Interestingly, no significant correlation between the overexpression of FGFR4 and OS was found in our analysis. Based on our previously published data on the prognostic role of FGFR4 in colorectal cancer and data on FGFR4 expression on esophageal squamous cell carcinoma, one would anticipate finding alike results in patients with adenocarcinomas of the esophago-gastric junction [41,44,47]. However, our findings are supported by our TCGA analysis of FGFR4 expression in patients with adenocarcinomas of the esophago-gastric junction, showing no significant correlation between expression and OS.

Even though the results of this study demonstrate that FGF8 and FGF18 are independent prognostic factors in patients with adenocarcinomas of the esophago-gastric junction, our study has certain limitations: one is the potential selection bias which was inevitably associated with only partial availability of tumor tissue, especially diagnostic biopsies before neoadjuvantly treated patients. Another limitation might be the retrospective nature of this single center research study. This is balanced by the fact that patient recruitment is ongoing and the patient database is maintained prospectively. Regarding the scoring method used in this study, one potential weak point has to be mentioned. Until now, no data exists on the expression of FGF8, FGF18, and FGFR4 on adenocarcinomas of the esophago-gastric junction investigated by immunohistochemistry and no signifier scoring method is available. Therefore, based on recently published recommendations for IHC scoring, conventional visual scoring based on our previously gained experiences using these antibodies on CRC tissue was used as appropriate [48,49,50]. However, further investigations on the expression of FGF8, FGF18, and FGFR4, using other methods including digital image analysis are urgently needed.

## 5. Conclusions

In conclusion, this is the first study that investigated the prognostic role of FGF8 and FGF18 including a subgroup analysis of neoadjuvantly treated and primarily resected patients using a preliminary TCGA analysis and immunohistochemistry demonstrating FGF8 and FGF18 as independent prognostic factors in resectable AEG. Furthermore, this is the first study comparing the expression of FGF8, FGF18, and FGFR4 in tumor tissue available before and after neoadjuvant treatment. However, due to the unexpected results of FGF18 overexpression and its protective nature, further investigations evaluating FGF8 and FGF18 as potential therapeutic targets are urgently needed.

## Figures and Tables

**Table 1:** FGF8, FGF18, and FGFR4 expression and their correlation with clinicopathologic parameters in patients with adenocarcinoma of the esophago-gastric junction.

Factors	FGF8					FGF18					FGFR4				
	high		low/absent		*p*-value	High		low/absent		*p*-value	high		low/absent		*p*-value
**Age (SD)**	65 (11)		65 (10)		>0.05	66 (12)		62 (10)		>0.05	66 (11)		64 (11)		>0.05
**Sex**					>0.05					>0.05					0.008
Male	75	(48.4%)	49	(31.6%)		38	(24.5%)	86	(55.5%)		74	(47.7%)	50	(32.3%)	
Female	19	(12.3%)	12	(7.7%)		11	(7.1%)	20	(12.9%)		10	(6.5%)	21	(13.5%)	
**Neoadjuvant treatment**					>0.05					>0.05					>0.05
Yes	37	(23.9%)	32	(20.6%)		26	(16.8%)	60	(38.7%)		31	(20.0%)	38	(24.5%)	
No	57	(36.8%)	29	(18.7%)		23	(14.8%)	46	(29.7%)		53	(34.2%)	33	(21.3%)	
**(y)pT**					0.003					>0.05					>0.05
0	0	(0.0%)	3	(1.9%)		0	(0.0%)	3	(1.9%)		0	(0.0%)	3	(1.9%)	
1	1	(0.6%)	2	(1.3%)		2	(1.3%)	1	(0.6%)		1	(0.6%)	2	(1.3%)	
2	17	(11.0%)	23	(14.8%)		11	(7.1%)	29	(18.7%)		22	(14.2%)	18	(11.6%)	
3	68	(43.9%)	32	(20.6%)		34	(21.9%)	66	(42.6%)		56	(36.1%)	44	(28.4%)	
4	8	(5.2%)	1	(0.6%)		2	(1.3%)	7	(4.5%)		5	(3.2%)	4	(2.6%)	
**(y)pN**					>0.05					>0.05					>0.05
0	17	(11.0%)	22	(14.2%)		16	(10.3%)	23	(14.8%)		18	(11.6%)	21	(13.5%)	
1	25	(16.1%)	13	(8.4%)		13	(8.4%)	25	(16.1%)		19	(12.3%)	19	(12.3%)	
2	24	(15.5%)	13	(8.4%)		7	(4.5%)	30	(19.4%)		25	(16.1%)	12	(7.7%)	
3	28	(18.1%)	13	(8.4%)		13	(8.4%)	28	(18.1%)		22	(14.2%)	19	(12.3%)	
**Tumor differentiation**					>0.05					>0.05					>0.05
0	0	(0.0%)	0	(0.0%)		0	(0.0%)	0	(0.0%)		0	(0.0%)	0	(0.0%)	
1	3	(1.9%)	4	(2.6%)		1	(0.6%)	6	(3.9%)		3	(1.9%)	4	(2.6%)	
2	29	(18.7%)	25	(16.1%)		20	(12.9%)	34	(21.9%)		26	(16.8%)	28	(18.1%)	
3	62	(40.0%)	32	(20.6%)		28	(18.1%)	66	(42.6%)		55	(35.5%)	39	(25.2%)	
**Lymph node ratio**					>0.05					>0.05					>0.05
<0.3	61	(39.4%)	42	(27.1%)		35	(22.6%)	68	(43.9%)		55	(35.5%)	48	(31.0%)	
≥0.3	33	(21.3%)	19	(12.3%)		15	(9.7%)	38	(24.5%)		29	(18.7%)	23	(14.8%)	
**R**				(0.0%)	>0.05					>0.05					>0.05
0	72	(46.5%)	51	(32.9%)		39	(25.2%)	84	(54.2%)		64	(41.3%)	59	(38.1%)	
1	22	(14.2%)	10	(6.5%)		10	(6.5%)	22	(14.2%)		20	(12.9%)	12	(7.7%)	
**UICC Staging**					0.01					>0.05					>0.05
0	0	(0.0%)	3	(1.9%)		0	(0.0%)	3	(1.9%)		0	(0.0%)	3	(1.9%)	
I	6	(3.9%)	9	(5.8%)		7	(4.5%)	8	(5.2%)		7	(4.5%)	8	(5.2%)	
II	10	(6.5%)	11	(7.1%)		10	(6.5%)	11	(7.1%)		12	(7.7%)	9	(5.8%)	
III	50	(32.3%)	24	(15.5%)		19	(12.3%)	55	(35.5%)		42	(27.1%)	32	(20.6%)	
IV	28	(18.1%)	14	(9.0%)		13	(8.4%)	29	(18.7%)		23	(14.8%)	19	(12.3%)	
**Mandard regression grade** *					0.039					>0.05					>0.05
1	0	(0.0%)	3	(1.9%)		0	(0.0%)	3	(1.9%)		0	(0.0%)	3	(1.9%)	
2	2	(1.3%)	1	(0.6%)		2	(1.3%)	1	(0.6%)		1	(0.6%)	2	(1.3%)	
3	7	(4.5%)	9	(5.8%)		7	(4.5%)	9	(5.8%)		7	(4.5%)	9	(5.8%)	
4	12	(7.7%)	14	(9.0%)		9	(5.8%)	17	(11.0%)		14	(9.0%)	12	(7.7%)	
5	16	(10.3%)	5	(3.2%)		5	(3.2%)	16	(10.3%)		9	(5.8%)	12	(7.7%)	
**Adjuvant Treatment**					>0.05					>0.05					>0.05
yes	45	(29.0%)	39	(25.2%)		24	(15.5%)	47	(30.3%)		42	(27.1%)	60	(38.7%)	
no	49	(31.6%)	22	(14.2%)		25	(16.1%)	59	(38.1%)		42	(27.1%)	11	(7.1%)	

FGF = fibroblast growth factor; FGFR = fibroblast growth factor receptor; SD = standard deviation; R = resection margin; UICC = Union for International Cancer Control; NT = neoadjuvant therapy; AT = adjuvant therapy * 1 = Complete regression; 2 = Presence of rare residual cancer cells; 3 = increase of number of residual cancer cells, but fibrosis still predominant 4 = residual cancer outgrowing fibrosis; 5 = absence of regressive changes.

**Table 2:** Univariable Cox regression analysis estimating the influence of FGF 8, FGF 18 and FGFR 4 expression and clinicopathological parameters on overall survival for patients with adenocarcinoma of the esophago-gastric junction.

	All Patients			Neoadjuvantly Treated Patients			Primarily Resected Patients		
*Factors*	Hazard Ratio	95% CI	*p* Value	Hazard Ratio	95% CI	*p* Value	Hazard Ratio	95% CI	*p* Value
**FGF 8** (ref.: high)									
low/absent	0.61	(0.43–0.87)	0.006	0.43	(0.27–0.83)	0.008	0.77	(0.48–1.24)	0.287
**FGF 18** (ref.: low/absent)									
high	0.66	(0.45–0.95)	0.027	0.54	0.30–0.97	0.039	0.80	(0.49–1.29)	0.363
**FGFR 4** (ref.: high)									
low/absent	0.9	(0.64–1.27)	0.562	1.05	0.61–1.79	0.871	0.89	(0.56–1.40)	0.615
**Age** (years)	1.00	(0.99–1.02)	0.887	0.98	0.96–1.01	0.217	1.01	(0.98–1.03)	0.341
**Sex** (ref. Male)									
female	0.87	(0.56–1.35)	0.529	1.27	0.62–2.63	0.511	0.89	(0.51–1.54)	0.672
**Neoadjuvant treatment** (ref.: no)									
yes	0.81	(0.57–1.14)	0.224	/	/	/	/	/	/
**(y)pT** (ref.: T3)									
0	0.22	(0.03–1.60)	0.135	0.22	(0.03–1.64)	0.139	/	/	/
1	0.17	(0.02–1.22)	0.080	0.16	(0.02–1.20)	0.075	/	/	/
2	0.58	(0.39–0.86)	0.008	0.63	(0.33–1.20)	0.160	0.54	(0.31–0.91)	0.020
4	2.74	(1.37–5.50)	0.005	29.63	(5.72–153.40)	<0.001	1.69	(0.72–3.99)	0.228
**(y)pN** (ref.: N3)									
0	0.23	(0.14–0.39)	<0.001	0.32	0.15–0.69	0.004	0.20	(0.10–0.39)	<0.001
1	0.40	(0.25–0.64)	<0.001	0.51	0.24–1.07	0.075	0.36	(0.19–0.68)	0.002
2	0.63	(0.40–1.00)	0.049	0.81	0.38–1.73	0.585	0.54	(0.31–0.97)	0.040
**Tumor differentiation** (ref.: 3)									
0 + 1	0.44	(0.18–1.08)	0.074	0.44	0.13–1.47	0.185	0.45	(0.11–1.87)	0.273
2	0.57	(0.39–0.83)	0.003	0.53	0.30–0.93	0.027	0.64	(0.39–1.06)	0.080
**Mandard regression grade** * (ref.: 3 + 4)									
1 + 2	/	/	/	1.40	0.49–4.02	0.529	/	/	/
5	/	/	/	2.28	0.76–6.86	0.143	/	/	/
**Lymph node ratio** (ref.: <0.3)									
≥0.3	1.93	(1.35–2.77)	<0.001	1.62	0.91–2.89	0.100	2.05	(1.29–3.25)	0.002
**R** (ref.: 0)									
1	2.06	(1.36–3.10)	<0.001	3.43	1.79–6.56	<0.001	1.51	(0.88–2.58)	0.134
**UICC Staging** (ref.: II + III + IV)									
0 + I	0.34	(0.19–0.63)	<0.001	2.91	1.14–7.43	0.026	0.34	(0.15–0.75)	0.007°
**Adjuvant treatment** (ref.: no)									
yes	1.55	(1.10–2.18)	0.013	1.33	0.75–2.38	0.330	1.58	(0.99–2.50)	0.052

CI = confidence interval; FGF = fibroblast growth factor; FGFR = fibroblast growth factor receptor; R = resection margin; UICC = Union for International Cancer Control; * 1 = Complete regression; 2 = Presence of rare residual cancer cells; 3 = increase of number of residual cancer cells, but fibrosis still predominant; 4 = residual cancer outgrowing fibrosis; 5 = absence of regressive changes; ° UICC I + II vs. III + IV in primarily resected patients.

**Table 3:** Multivariable Cox regression analysis estimating the influence of FGF 8, FGF 18 and FGFR 4 expression on overall survival for patients with adenocarcinoma of the esophago-gastric junction.

	All Patients			Neoadjuvantly Treated Patients			Primarily Resected Patients		
Factors	Hazard Ratio	95% CI	*p* Value	Hazard Ratio	95% CI	*p* Value	Hazard Ratio	95% CI	*p* Value
**FGF 8** (ref.: high)									
low/absent	0.68	(0.46–0.99)	0.042	0.43	(0.22–0.82)	0.011	1.04	(0.63–1.72)	0.882
**FGF 18** (ref.: low/absent)									
high	0.71	(0.48–1.04)	0.08	0.44	(0.22–0.86)	0.017	0.81	(0.49–1.33)	0.408
**FGFR 4** (ref.: high)									
low/absent	1.04	(0.72–1.50)	0.834	1.02	(0.58–1.81)	0.945	1.03	(0.63–1.67)	0.908

CI = confidence interval; FGF = fibroblast growth factor; FGFR = fibroblast growth factor receptor.
